# Unravelling the mosaic: Epigenetic diversity in glioblastoma

**DOI:** 10.1002/1878-0261.13706

**Published:** 2024-08-15

**Authors:** Sara Lucchini, Myrianni Constantinou, Silvia Marino

**Affiliations:** ^1^ Brain Tumour Research Centre, Blizard Institute, Faculty of Medicine and Dentistry Queen Mary University of London UK; ^2^ Barts Brain Tumour Centre, Faculty of Medicine and Dentistry Queen Mary University of London UK; ^3^ Barts Health NHS Trust London UK

**Keywords:** brain tumours, cell‐of‐origin, epigenetics, glioblastoma, interpatient heterogeneity

## Abstract

Glioblastoma is the most common primary malignant brain tumour. Despite decades of intensive research in the disease, its prognosis remains poor, with an average survival of only 14 months after diagnosis. The remarkable level of intra‐ and interpatient heterogeneity is certainly contributing to the lack of progress in tackling this tumour. Epigenetic dysregulation plays an important role in glioblastoma biology and significantly contributes to intratumour heterogeneity. However, it is becoming increasingly clear that it also contributes to intertumour heterogeneity, which historically had mainly been linked to diverse genetic events occurring in different patients. In this review, we explore how DNA methylation, chromatin remodelling, microRNA (miRNA) dysregulation, and long noncoding RNA (lncRNA) alterations contribute to intertumour heterogeneity in glioblastoma, including its implications for advanced tumour stratification, which is the essential first step for developing more effective patient‐specific therapeutic approaches.

## Graphical abstract

Glioblastoma is a malignant brain tumour characterised by prominent heterogeneity at both the intra‐ and interpatient level. Despite advances in the genetic and transcriptional stratification of patients, clinical outcomes have not improved. Here we review epigenetic dysregulation in glioblastoma, focusing on its contribution to interpatient heterogeneity with an emphasis on classification refinement and novel therapeutic approaches.

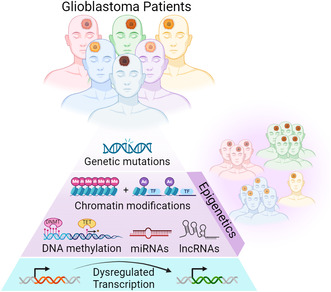

**Abbreviations**
5caC: 5‐carboxylcytosine
5fC: 5‐formylcytosine
5hmC: 5‐hydroxymethylcytosine
5mC: 5‐methylcytosine
AER: active enhancer region
APCs: astrocyte progenitor cells
ARE: active regulatory elements
BBB: blood brain barrier
ceRNA: competing endogenous RNA
CL: classical – transcriptional subtype
CNS: central nervous system
CpG site: cytosine preceding a guanine nucleotide
CUT&RUN: cleavage under targets and release using nuclease
CUT&Tag: cleavage under targets and tagmentation
DGIdb: drug–gene interaction database
DIPG: diffuse intrinsic pontine gliomas
DNMT: DNA methyltransferase
EMT: epithelial‐to‐mesenchymal‐transition
EOR: extent of resection
EPSC: expanded potential stem cells
FFPE: formalin fixed paraffin embedded
GBO: glioblastoma organoids
GICs: glioblastoma initiating cells
GLICO: glioblastoma cerebral organoid
GSCs: glioblastoma stem cells
GSEA: gene set enrichment analysis
IDHmut: IDH mutant
irlncRNA: immune‐related lncRNA
Lmce: lncRNA‐mediated ceRNA
lncRNA: long noncoding RNAs
MB: medulloblastoma
MES: mesenchymal – transcriptional subtype
MGMT: O6‐methylguanine DNA methyltransferase
miRNA: microRNA
MR: master regulators
NGS: next‐generation sequencing
NPCs: neural progenitor cells
OPCs: oligodendrocyte progenitor cells
OS: overall survival
PDX: patient‐derived xenograft
PFS: progression free survival
PN: proneural – transcriptional subtype
pri‐miRNA: primary miRNAs
PTM: post‐translational modifications
RRBS: reduced representation bisulphite sequencing
SAM: S‐adenyl methionine
SE: super‐enhancer
SYNGN: syngeneic comparison of GIC and iNSC
taTRE: tumour‐associated transcriptional regulatory elements
TET: ten‐eleven translocation
TF: transcription factor
TME: tumour microenvironment
TMZ: temozolomide
TRE: transcriptional regulatory elements
WGBS: whole genome bisulphite sequencing

## 1 Introduction

Glioblastoma is the most common and aggressive primary brain tumour, with a median survival of only 14 months in patients receiving the standard of care treatment, which includes maximal safe surgical resection followed by radio‐ and chemotherapy [1]. Personalised mutation‐based therapeutic approaches have been trialled, although they have failed to improve survival to date [2]. This outcome is a consequence of several factors, including its location within the brain rendering the tumour poorly accessible at surgery, its highly infiltrative growth pattern [3], and the presence of the blood–brain barrier (BBB), which interferes with drug delivery [4]. Moreover, the remarkable intra‐ and intertumour molecular heterogeneity is certainly one of the main challenges of this disease. Historically, intertumour heterogeneity in glioblastoma, i.e., the presence of tumour cells with unique characteristics in the tumour of each patient, has been ascribed mainly to diverse genetic events leading to the formation of this tumour in different patients. The best‐characterised genetic lesions involved in glioblastoma pathogenesis are summarised in the 2021 WHO classification of tumours of the central nervous system (CNS): deletions or inactivation of *TP53*, *PTEN*, *NF1,* and *CDKN2A/B*; amplification of *CDK4/6*, *EGFR,* and *PDGFRA*; mutations of the *TERT* promoter and copy number changes at chromosome 7 (amplifications) and 10 (deletions) [5]. Additional genes with significant mutation frequency were also identified when analysing the whole exome of 291 glioblastoma patients, including *SPTA1*, *ATRX,* which is a member of the SWI/SNF family of chromatin remodellers, *GABRA6*, *KEL*, leucine‐zipper‐like transcriptional regulator 1, or *LZTR1* [6]. Moreover, the analysis of DNA copy number variation of 543 glioblastoma samples shed light on some common amplification events on chromosome 7 (*EGFR*, *MET*, *CDK6*), chromosome 12 (*CDK4* and *MDM2*), and chromosome 4 (*PDGFRA*). Additional gains that are frequently found affect *SOX2*, *CCND1* and *CCND2*, and *MYCN*. For an extensive analysis of the mutational burden of more than ten thousand gliomas, please see Touat *et al*. [7], as a comprehensive summary of all genetic lesions found in glioblastoma is beyond the scope of this review. Importantly, IDH‐mutant (IDHmut) high‐grade gliomas are no longer called glioblastoma in the 2021 WHO classification of CNS tumours [5], hence contributing to removing an element of confusion in the definition of the tumour on a genetic basis.

It is increasingly evident, though, that characterising the genetic events underpinning glioblastoma development is not sufficient to entirely understand its heterogeneity and dismal outcome. The contribution of epigenetic deregulation to glioblastoma heterogeneity has been studied in the context of its intratumour component, i.e., the multiple different cellular and molecular populations within a single tumour [8, 9, 10, 11] and will not be the focus of this review. Here, we will review the role of epigenetic deregulation in glioblastoma, with a particular focus on its contribution to intertumour heterogeneity, including its impact on stratification, identification of novel therapeutic targets [12, 13, 14], and improved modalities to enable the future matching of available drugs to patient subgroups for personalised therapies. It is important to note that the inclusion of the now called astrocytoma, IDHmut, CNS WHO grade 4 in many pre‐2021 studies may have skewed to a certain extent the overall results. Caution is needed when interpreting these data, although this is felt to be of modest bearing in the majority of cases where the results have now been validated in glioblastoma‐only cohorts.

## 2 Epigenetic dysregulation contributes to glioblastoma heterogeneity

**Fig. 1:**
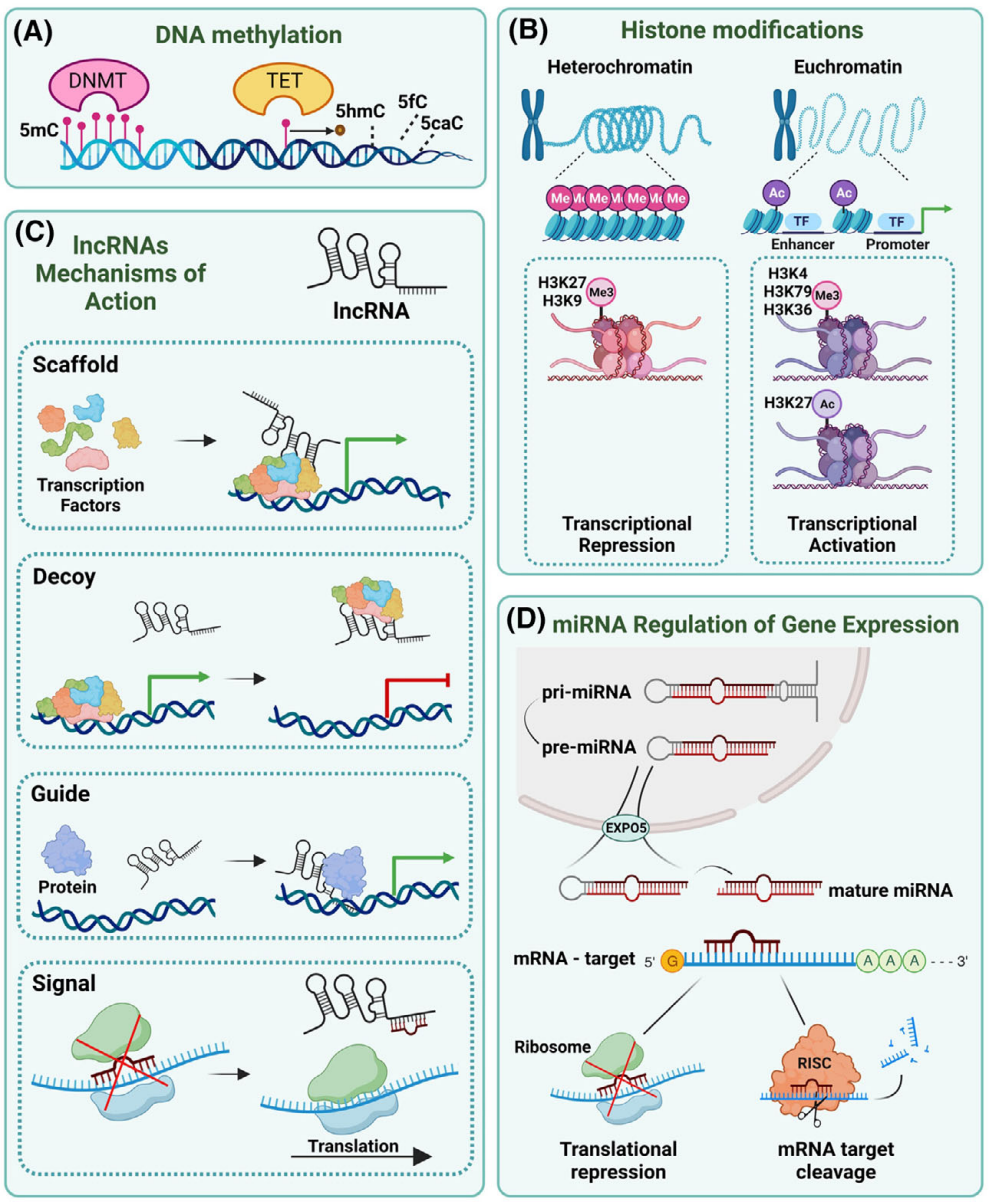
Epigenetic players and their mechanisms of action. (A) Schematic of DNA methylation and demethylation as mediated by two enzymes: DNA methyltransferase (DNMT, catalysing the addition of a methyl group to the 5′ carbon of a cytosine base in CpG dinucleotides) and ten‐eleven translocation (TET, oxidising 5‐methylcytosine leading to DNA demethylation). (B) Open and closed chromatin conformations, euchromatin, and heterochromatin, respectively, are mediated by post‐translational modifications of histones. In this schematic, the most common histone modifications playing a role in repressing or activating transcription are represented. (C) Representation of the main functions of long noncoding RNAs (lncRNAs) involved in both transcription and translation regulation. lncRNAs regulate gene expression via four main mechanisms: acting as scaffolds, they bring essential transcription factors to the target DNA region; functioning as decoys, they remove the transcription machinery from the promoter region; lncRNAs can act as guides, bringing chromatin modifying complexes to the DNA target region; or as molecular signals, interfering with miRNAs. (D) Schematic of microRNAs (miRNAs) production process and their mechanism of repressing translation, by binding to mRNA targets and consequently blocking the ribosome binding, or by binding an RISC complex and bringing it to the RNA target, leading to its cleavage.

Epigenetics encompasses a spectrum of molecular modifications of the DNA that regulate the expression and activity of genes, without altering the underlying DNA sequence [15]. Four main regulatory mechanisms underpin these modifications (Fig. 1): DNA methylation, chromatin remodelling, microRNAs (miRNAs), and long noncoding RNAs (lncRNAs); they are mitotically and meiotically inheritable and play a crucial role in maintaining genomic stability [16, 17]. Because of the impact on gene expression of these regulatory processes, they play a crucial role in tumorigenesis when disrupted [18]. Here we describe and critically analyse the existing literature about these four main regulatory mechanisms in glioblastoma, particularly focusing on how they impact intertumour heterogeneity. As epigenetic changes are reversible, an in‐depth characterisation of the disrupted epigenetic landscape in these tumours can identify novel biomarkers and druggable targets for cancer therapy.

### 2.1 DNA methylation

DNA methylation occurs on a cytosine preceding a guanine nucleotide (CpG site). Two major enzyme families regulate the process: the DNA methyltransferases (DNMTs) and ten‐eleven translocation (TET) methylcytosine dioxygenases (Fig. 1A). DNMTs catalyse the addition of a methyl group from S‐adenyl methionine (SAM) to the 5′ carbon of a cytosine base in CpG dinucleotides, resulting in the formation of 5‐methylcytosine (5mC), which leads to compaction of the chromatin structure, hence restricting transcription factors accessibility to their binding sites. DNA demethylation is catalysed by TET proteins, which revert the above‐mentioned condensation by oxidising 5‐methylcytosine (5mC) to 5‐hydroxymethylcytosine (5hmC), 5‐formylcytosine (5fC), and 5‐carboxylcytosine (5caC) [19, 20]. Since 40% of CpG islands (stretches of ~300–3000 base pairs with high CpG content) reside within or near mammalian gene promoters, and 50% of these contain transcription start sites [21, 22, 23], the resulting impact of aberrant DNA methylation on transcription can be significant. Several methods have been developed to analyse genome‐wide DNA methylation patterns. The EPIC array relies on multiplexed genotyping of bisulphite‐converted genomic DNA and covers 935 000 CpG sites (HumanMethylationEPIC version 2.0) [24]. It is currently widely used, as it is easy to carry out, including on formalin‐fixed paraffin‐embedded (FFPE) material, which makes it amenable to a clinical setting, where patient tumour samples are predominantly stored as FFPE material. Whole‐genome bisulphite sequencing (WGBS) is based on sodium bisulphite treatment of genomic DNA, which is then sequenced at a single nucleotide resolution with 95% coverage of all CpGs in the human genome (2 CpG/100 bp) [25]. Reduced representation bisulphite sequencing (RRBS) exploits a methylation‐sensitive enzymatic digestion of unmethylated DNA, which results in the enrichment of GC‐rich (methylated) areas of the genome, allowing more than three CpG/100 bp to be captured.

Early studies on cancer epigenome revealed a global hypomethylation bias, which was responsible at least in part for the transcriptional activation of oncogenes; however, hypermethylation of the CpG island at tumour suppressor genes and homeobox genes has also been described [26, 27, 28, 29, 30, 31].

**Fig. 2:**
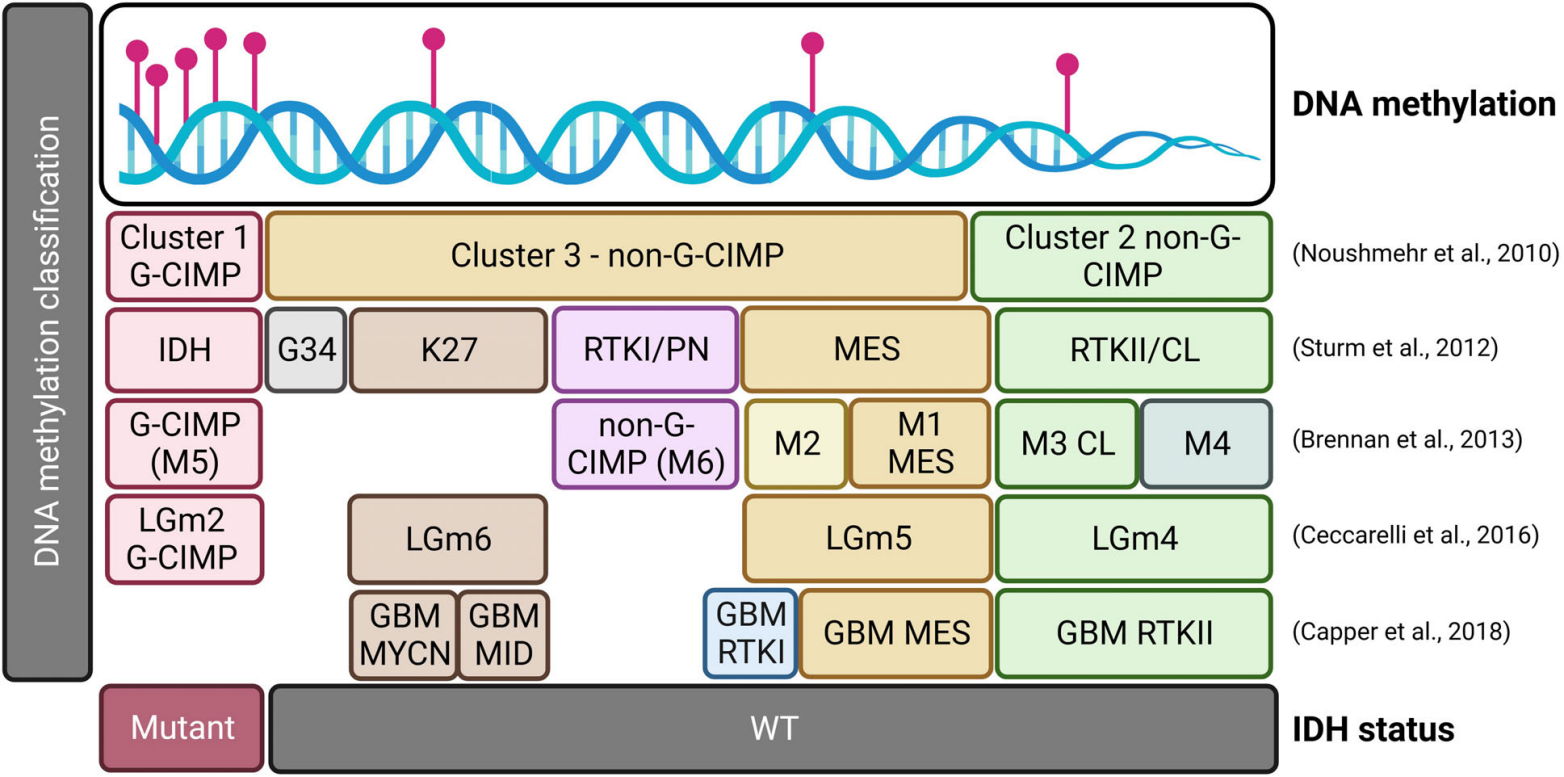
DNA methylation‐based high‐grade glioma classification. The schematic depicts the relative overlap between subtypes reported in the indicated studies on the right. The hypermethylation signature (G‐CIMP, M5) is associated with the IDH1 mutants, which is now an independent tumour entity referred to as astrocytomas IDH‐mutant CNS WHO grade 4, while the non‐G‐CIMP (hypomethylation) is associated with wildtype (WT) IDH status. Non‐G‐CIMP WT‐IDH glioblastoma can be further subclassified into three main adult glioblastoma classes, RTKI, RTKII, and MES (mesenchymal), and adolescent/paediatric gliomas K27 and G34, based on DNA methylation patterns of methylated/unmethylated probes and copy number alterations, with similarities and differences among the different classification methods. M1 and M2 were associated with MES, M3, and M4 with RTKII/CL (classical) and M6 with RTKI/PN (proneural). The TCGA classes LGm1–6 described by Ceccarelli *et al*. [43] recapitulated specific classes identified in the reference cohort used in the DKFZ classifier by Capper *et al*. [49].

In glioblastoma, hypermethylation at the O6‐methylguanine DNA methyltransferase (*MGMT*) gene promoter is currently the most relevant biomarker predicting response to alkylating drugs [32, 33, 34]. Tumours with an unmethylated *MGMT* promoter region, where *MGMT* is active, have brisk DNA repair mechanisms and are less sensitive to cytotoxic treatment with temozolomide (TMZ), which remains the most effective drug in this tumour. Moreover, hypermethylation of the CpG island in promoters of genes involved in signalling pathways often deregulated in these tumours have been reported, with *RB1* methylation observed in 14% of glioblastomas analysed (5 of 35), *PTEN* promoter methylation in 35% (27 of 77) glioblastoma patients' samples, and *TP53* promoter methylation in 21.4% (9 of 42) glioblastoma [35, 36, 37, 38, 39, 40]. Importantly, assessment of the global DNA methylation profile of 272 TCGA high‐grade gliomas led to the identification of three distinct clusters: Cluster 1 was enriched for the proneural (PN) transcriptional subtype and also associated with IDH mutations and longer survival. Cluster 2 and Cluster 3 were mainly enriched for the classical (CL) and mesenchymal (MES) glioblastoma subgroups, respectively [41]. Integration of these DNA methylation profiles with genetic and gene expression led to the consolidation of the three subgroups, defined in this study as RTK I (Proneural, *PDGFRA*), MES (mesenchymal), and RTK II (classical, *EGFR*) [42]. Clustering of 396 high‐grade gliomas confirmed these three subtypes [6], here defined Cluster M1 (with 60% enrichment for MES), M3 (58% enrichment for CL), and M5 (mainly enriched for PN glioblastoma, but also including IDH‐mutant tumours, now referred to as astrocytoma IDHmut CNS WHO grade 4 in the 2021 WHO classification) (Fig. 2). Additionally, cluster M2 was associated with MES, Cluster M6 assigned to RTKI/PN but IDH1‐wildtype and with concurrent *PDGFRA* copy number alterations and Cluster M4 was assigned to RTKII/CL. In addition, M2, M4, and M6 were enriched for mutations of chromatin modifier genes, with M2 showing missense mutations and deletions of *MLL* or *HDAC* genes. Similar results were obtained also when analysing the DNA methylation profiles of 932 gliomas [43]. Interestingly, the functional validation of IDH‐mutant tumours as a subgroup with unique biology, contributed to their classification as an independent tumour entity in the new 2021 WHO classification.

Subgrouping glioblastomas on the basis of transcriptional profiling has been hampered by the significant intratumour spatial heterogeneity, with more than one subtype detected within one single bulk tumour sample and frequent changes in subgroup allocation at recurrence. Importantly, very low true spatial heterogeneity of DNA methylation subtypes was observed within 27 individual tumours, where samples from different regions of each tumour were available (16 in their cohort, 11 from a second cohort) [44]. Here, most of the detected intratumor heterogeneity at the DNA methylation level was due to the low tumour purity of the samples. In fact, after accounting for the tumour purity, no significant spatial heterogeneity was observed in the main classes. Nevertheless, temporal switches have also been described for the DNA methylation subgrouping, together with the identification of master regulators (MRs), which are able to predict transitions in glioblastomas [45]. This is in keeping with a previous study describing *SOX10* as hypomethylated and overexpressed in RTKI glioblastoma subtype, with its repression leading to a MES phenotype transition [46]. Moreover, a recent study of 345 patients showed a 23.8% switch in DNA methylation subclasses upon disease progression in longitudinal samples of 22 patients [47] as opposed to 49% transcriptional subtype switch observed at recurrence [48].

DNA methylation profiles were also leveraged to develop a classifier of brain tumours, the DKFZ classifier (http://www.molecularneuropathology.org/), which is now one of the test modalities used to reproducibly classify CNS tumours, in agreement with the 2021 WHO classification, with high confidence within a clinically relevant timeline [49, 50]. Using a random forest algorithm with multiple decision trees and incorporating DNA methylation signatures based on 32 000 most variably methylated probes (probe selection) identified from a reference cohort, the authors developed a useful tool, which in conjunction with histological assessment, advances the classification of these neoplasms. The classifier has recently been shown to lead to refinement of diagnosis in 84% of the cases and it has impacted the treatment decision in 15% of patients [51] in a cohort of 55 CNS tumours that were otherwise challenging to classify. In glioblastoma, the DKFZ CNS tumour classifier has identified new subclasses, which now include RTK1, RTK2, mesenchymal subtype, mesenchymal subtype subclass B, primitive neuronal compartment, and subtype posterior fossa [52, 53].

DNA methylation profiles have also been utilised to infer the composition of the tumour microenvironment (TME), to distinguish samples with high and low necrosis, and to predict immune cell infiltration in the tumour, as well as high and low proliferation rates of the tumour cells [54]. An association between DNA methylation subtypes and CD3^+^/CD8^+^ T‐cell infiltration was also observed, with MES exhibiting the highest infiltration of immune cells. The amount of CD3^+^ T‐cell infiltration inversely correlated with the overall survival (OS), with the lowest survival of 15.5 months in the MES, 16.0 in the RTKI, and 24.0 in RTKII group [55], although it remains unclear whether the presence of infiltrating T‐cells has an antitumour or rather an immunosuppressive protumour role with both positive [36, 56, 57, 58] negative [59], or absence of correlation [60] previously reported.

The impact of DNA methylation subgrouping of glioblastoma on patient management is a promising emerging field of research, with studies reporting DNA methylation patterns as able to distinguish patients with the longest and shortest survival [41, 45, 48, 54, 55]. The RTKII methylation subtype has been described as an indicator for preoperative and postoperative seizures, hence raising the possibility that patients of this subtype may benefit from an antiepileptic treatment [61]. Furthermore, overall survival (OS) and progression‐free survival (PFS) of patients in the RTKI and RTKII subclasses have been shown to be significantly longer based on the extent of resection (EOR) they received, while patients of the MES subclass did not gain a significant benefit from EOR [48]. Finally, DNA methylation analysis on primary patients' samples also proved useful in predicting changes at recurrence. Spatiotemporal DNA‐methylation patterns of 200 biopsies, derived from 77 patients, revealed prediction of recurrence based on DNA methylation biomarkers identified at the time of primary diagnosis [45].

### 2.2 Post‐translational modifications of histones and chromatin remodelling

Histones are the basic units of chromatin. These proteins have (C)‐terminal and (N)‐terminal residues extruding the octamer structure, which can be post‐translationally modified when containing lysine, arginine, serine, and threonine [62]. Post‐translational modifications (PTMs) of these residues regulate gene expression by influencing chromatin accessibility, and they are controlled by three main categories of enzymes: ‘writers’, ‘erasers’, and ‘readers’ [63]. Dysregulation of these enzymes can lead to transcriptional abnormalities, which have been implicated in the development and progression of several diseases including glioblastoma.

Histone PTMs can be activating or repressing gene expression (Fig. 1B). The most common PTMs associated with a transcriptionally active chromatin are trimethylation of H3 at lysine 4 (H3K4me3), lysine 79 (H3K79me3), and lysine 36 (H3K36me3), which promote an accessible structure (euchromatin) that allows the transcriptional machinery enzymes to bind to the DNA [64]. Furthermore, acetylation of N‐terminal lysine residues of histones H3 and H4 (such as H3K27ac) promotes an open chromatin structure by neutralising the positive charges and reducing the affinity of histones with the negatively charged DNA [65]. In contrast, methylation of lysines 9 and 27 of histone H3 (H3K9me3 and H3K27me3, respectively) are associated with a more condensed, hence inactive chromatin structure because of the reduced DNA accessibility of the transcription machinery (heterochromatin) [66]. The chromatin structure is also influenced by the so‐called transcriptional regulatory elements (TRE), regions of the genome including promoters, enhancers, and insulators. TREs regulate gene expression by facilitating or inhibiting chromatin decompaction and transcription initiation [67]. Depending on the chromatin state, transcription factors (TFs) can regulate gene expression by engaging with nucleosomes and binding to enhancers, cis‐regulatory elements, thus recruiting coactivators of the transcription machinery and RNA Polymerase II to the respective target genes. Some of them are known as ‘master’ TFs. They can not only bind to their own enhancer region, but also to larger enhancers that regulate other ‘master’ TFs, forming a network of autoregulatory circuitry. These larger enhancers are called super‐enhancers (SEs) and their identification and epigenetic regulation can help to uncover key regulatory mechanisms in complex gene expression networks [68].

ChIPseq and ATACseq are primarily used for studying DNA–protein interactions and the chromatin accessibility landscape, respectively [69]. ChIPseq combines chromatin immunoprecipitation with next‐generation sequencing (NGS) to investigate the interactions between specific transcription factors, other chromatin‐related proteins, or histone tail modifications with the DNA [70, 71]. Recently, two more efficient chromatin profiling techniques, which not only require low cell numbers but are also characterised by a high signal‐to‐noise ratio, have been developed: CUT&RUN (Cleavage Under Targets and Release Using Nuclease) [72] and subsequently CUT&Tag (Cleavage Under Targets and Tagmentation) [73, 74]. Both these strategies are based on enzyme tethering *in situ*. The chromatin proteins or regions of interest are targeted by a fusion protein and the underlying region is released thanks to an enzymatic reaction. ATAC‐seq is used to assess chromatin accessibility on a genome‐wide scale. It exploits a hyperactive Tn5 transposase, which preferentially inserts itself into accessible regions of the genome and not only cleaves double‐stranded DNA but also tags the fragments with sequencing adaptors. These DNA fragments, representing the open and accessible chromatin, are then sequenced, allowing for the identification of regions with increased or decreased accessibility [75, 76].

Several aberrations in the activation or inactivation of epigenetic‐related enzymes together with chromatin remodelling genes have been shown in glioblastoma [77, 78]. *SOX10,* for example, is an RTKI‐specific MR. [46] MRs are proteins known to regulate and define cellular states in cancer [79, 80], and they have been described to be subtype‐specific in glioblastoma. Alteration of this machinery can lead to the transition between subtypes. *SOX10* repression induces a remodelling of the enhancers landscape, which triggers the subtype transition from RTKI to the mesenchymal subtype.

Integrative analysis of PTM profiles with whole‐exome profiles, copy number variation, and gene expression has advanced our understanding of the contribution of chromatin regulation to intertumour heterogeneity in glioblastoma. The first such study compared the SE landscape of 30 primary glioblastoma initiating / stem cells (GICs) cultures by performing a hierarchical clustering of H3K27ac activity and found two significantly different SE states defining two subgroups of GICs. This differential analysis revealed the SEs driving group‐specific differences, which were 597 and 651 SEs for *Group 1* and *Group 2,* respectively. Employing single‐sample gene set enrichment analysis (GSEA) [81], the two groups have been further characterised using TCGA‐defined molecular signatures from bulk primary glioblastomas. *Group 1* displayed ‘mesenchymal’ features and *Group 2* both ‘proneural’ and ‘classical’ features, which usually appear distinct on transcription profiling only. *Group 1* was associated with overexpression of *OLIG2* at the mRNA level together with enriched acetylation of H3K27 at the *OLIG2* locus, while *Group 2* was associated with enriched H3K27ac on SEs regulating the JAK/STAT pathway [82]. In another study, colocalisation of H3K27ac and H3K4me1 identified 1817 ‘common enhancers’ that were shared at least among three out of 11 bulk tumour samples. Of these 1817, 307 were found to be enriched for pathways mediating cell–cell interactions. Subtype‐specific enhancer states were also identified; however, in contrast to the previous study, genes expressed in MES/CL were found to be poorly expressed in PN and vice versa [8].

**Fig. 3:**
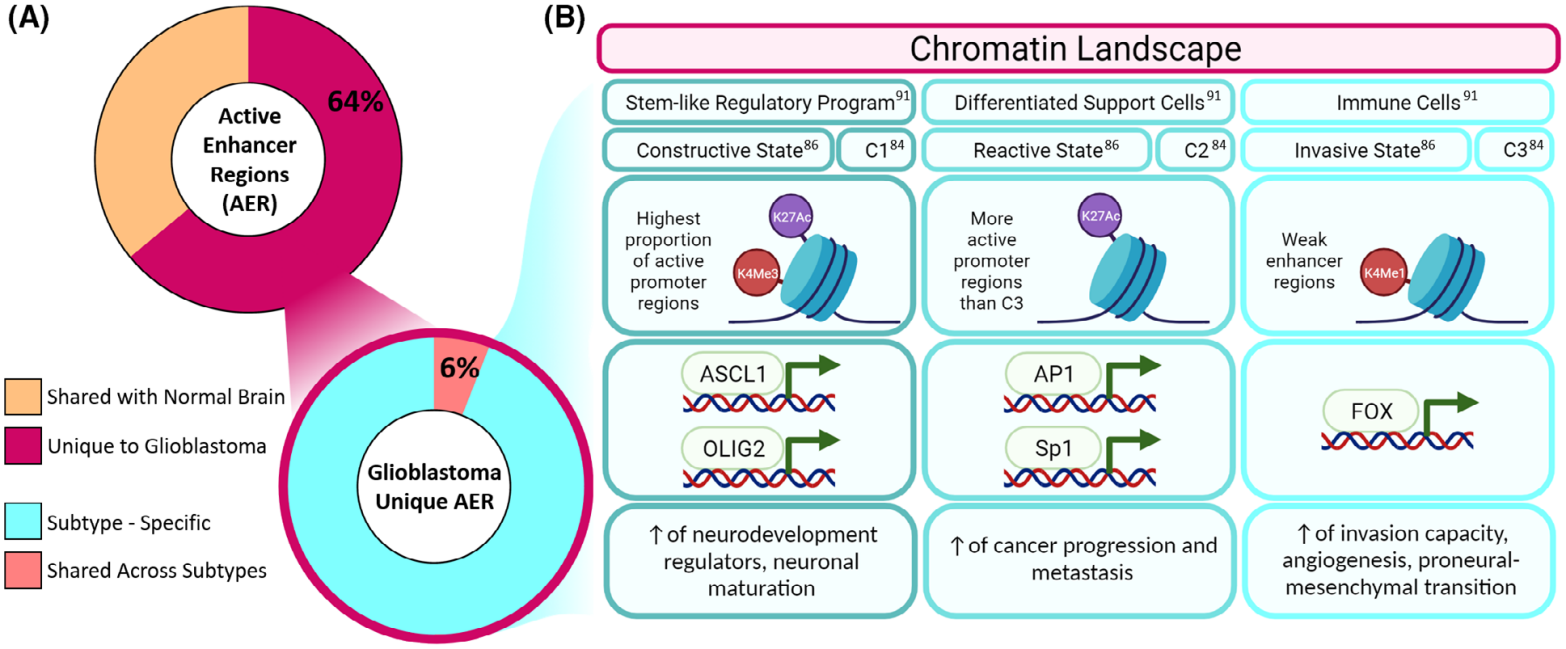
Chromatin landscape of the three glioblastoma subtypes. (A) Distribution of active enhancer regions in glioblastoma. 64% is not shared with normal brain tissue; of these, only 6% is shared across patients, with the remaining 94% of glioblastoma‐related AERs (active enhancer region) being subtype‐specific. (B) The chromatin landscape of glioblastoma can be subdivided into three classes (columns), as shown in three different studies. Even though the three studies named the subclasses differently (first row), the main features are shared, in particular, specific PTM (post‐translational modifications) of histones (second row), enrichment of specific transcription factors (third row), and consequent upregulation of specific biological processes (fourth row).

ATAC‐Seq of 60 patient‐derived GICs lines allowed to robustly distinguish three clusters of GICs, referred to as *C1*, *C2,* and *C3*. *C1* is characterised by the highest proportion of active promoter regions (H3K27ac and H3K4me3) and it contains the majority of proneural and classical lines. *C2* has a higher proportion of active regions (H3K27ac) as compared to *C3*, which is characterised by weak enhancer regions (H3K4me1), and these two subgroups comprise the mesenchymal lines. Moreover, the integration of these data with GICs single‐cell gene expression data [83] suggested that the three clusters represent a gradient of states from the C1‐progenitor cell‐like state (highest expression of NPC1, NPC2, and OPC genes) to *C2*‐mesenchymal‐like state (high expression of MES1 and MES2 genes and the lowest expression of NPC1, OPC, and AC genes) via the *C3*‐intermediate state [84]. Three states were identified also in another study, which derived ATAC‐Seq profiles from 27 GIC lines [85]. Integration of the two datasets allowed identifying genes that are exclusively enriched for both promoter chromatin accessibility and gene expression and exploit GSEA to define the clusters as *reactive state*, *constructive state,* and *invasive state* [86]. Interestingly, these states correlate with the previously described *C2*, C1, and *C3* states [84], respectively, when considering the enriched cluster‐specific TFs motifs. *C1* and *constructive states* are both enriched for neural development regulators such as ASCL1 [87] and OLIG2 [88]. *C2* and *reactive state* are enriched for motifs involved in cancer progression and metastasis, AP‐1 complex [89] and SP1 regulatory network [90], respectively. Finally, *C3* and *invasive state* show enrichment of Forkhead‐box (FOX) TFs, which are related to invasion capacity, angiogenesis, lower survival, and tumorigenicity of MES GICs (Fig. 3).

Three states have also been detected when analysing the regulatory programs defined by tumour‐associated transcriptional regulatory elements (taTREs) in 20 primary glioblastoma samples, which were described as *stem‐like regulatory program*, *differentiated support cells,* and *immune cells* [91]. The TF motifs enriched in each state are concordant with those previously described. The *stem‐like regulatory program* is enriched for POU‐domain‐containing TFs, such as POU3F2, which is involved in neural‐development and drives neuronal maturation in combination with ASCL1 [92]. The *differentiated support cells* showed enrichment for AP‐1 and the *immune cells* are enriched for the NF‐kB family, which not only has a role in inflammatory response [93], but is also activated, together with FOXD‐ALDH1A3, in MES GICs and during the proneural‐mesenchymal transition [94] (Fig. 3).

An additional level of glioblastoma heterogeneity is provided by the study of active regulatory elements (ARE) in 95 glioblastoma biopsies, 12 normal brain samples, and 38 glioblastoma cell lines followed by the integration of the results with the gene expression‐based glioblastoma classification [95]. In this study, four molecular subgroups were identified: *AC1‐MES*, *AC1‐CL*, *AC2‐PN*, and *AC3‐PN*. Interestingly, *AC1‐MES* and *AC1‐CL* have similar SE topography and ARE architecture, although showing different gene expression profiles and different pathways enrichment. In contrast, the ARE topography of *AC2‐PN* and *AC3‐PN* subgroups strongly differ but their gene expression profile is similar to the proneural signature. This underscores the limitations of relying solely on expression‐based classification for glioblastoma subtypes, as it does not entirely capture the intricate biology of these tumours. Incorporating epigenetic regulations into the analysis is indeed providing an additional layer of information, enhancing our understanding of the underlying complexities. In addition, they show a link between glioblastoma‐associated SEs and neuro‐developmental enhancers, whereby the expression of oncogenic TFs is enabled, raising the possibility that the SEs landscape is reshaped in the cells of origin to promote tumourigenesis.

Taken together, these studies suggest that the chromatin landscape defines interpatient heterogeneity in glioblastoma. In fact, 64% of tumour active‐enhancer regions are unique to glioblastoma when compared to normal brain and, of these, only 6% is shared across all subtypes, while the rest is subtype‐specific [46]. In addition, the chromatin accessibility landscape of 60 patient‐derived GIC lines shows a high degree of interculture heterogeneity: 25% of the ATAC peaks are culture‐unique, while only 1.5% are shared among all 60 patients. This suggests that the developmental regulation inherited from the cell of origin is the primary determinant of cellular states and contributes to epigenetic heterogeneity [84]. Moreover, scATACseq and trajectory analysis of glioblastoma cerebral organoid (GLICO) cocultured with five patient‐derived GSC lines, have shown that patient‐specific differences in chromatin accessibility and transcriptomic signatures are mainly found in the differentiated‐like states. In contrast, early stem‐like states share similar trajectories in all five patients [96], possibly resembling the cells of origin and raising the possibility they may be attractive therapeutic targets.

### 2.3 miRNA

MiRNAs, short for microRNAs, are a class of small noncoding RNA molecules that play a crucial role in the regulation of gene expression (Fig. 1D). These molecules are transcribed in a longer preprocessed form known as primary miRNAs (pri‐miRNAs), which are processed in the nucleus by RNase III, resulting in mature miRNAs that are typically 20–22 nucleotides in length [97]. Mature miRNAs form hairpin loop structures, which enable them to interact with mRNA molecules by partially binding to their complementary sequence [98]. This interaction can lead to the deregulation of related mRNAs, the inhibition of targeted genes, and even the suppression of the translation process. Moreover, each miRNA can target several mRNAs and several miRNAs (sharing the same basic sequence) can bind to the same target mRNAs [99]. Due to their high modulatory potential, miRNAs have been shown to regulate over 25% of expressed genes in humans [100]. Regulation of miRNAs is due to their transcription unit, dysregulation of which impacts gene expression.

The remarkably informative nature of these small molecules in a tumour context, including reflecting the differentiation state and developmental lineages of tumours, has been known for over two decades [101]. In the brain, they influence cell identity and, consequently their altered regulation contributes to tumours development, including glioblastoma [102], where they contribute to the deregulation of pathways controlling cell cycle, cell death, and inflammation, such as the *PI3K‐AKT‐PTEN* pathway [97].

MiRNA expression‐based clustering has been suggested to provide a more accurate sample classification, both in terms of histology and prognosis, than the clustering based on mRNA expression [103]. MiRNA expression profiles of 261 glioblastomas from TCGA were screened based on 121 previously selected miRNAs and consensus clustering identified five subgroups, which correlated with differentiation‐related mRNA signatures [104]. *Subclass I* was defined by the expression of oligo‐neural precursors miRNA cluster; *subclass II* by increased expression of astrocytic, oligo‐neural, and multipotent precursor clusters; *subclass III* by increased expression of the neuronal precursors cluster; *subclass IV* by increased expression of the neuro‐mesenchymal precursors; and *subclass V* by increased expression of the astrocytic cluster. Therefore, the authors defined the five glioblastoma subclasses respectively as: *oligoneural*, *radial glial*, *neural*, *neuromesenchymal,* and *astrocytic*. When comparing these with the ones based on gene expression [105], an enrichment of the miRNA‐based *oligoneural*, *radial glial,* and *astrocytic* groups is found in the proneural, classical, and mesenchymal mRNA‐based subgroups, respectively. Notably, when miRNA expression was used, 20–50% of tumours in the four subclasses were reclassified. Additionally, it was found that both the *neural* and *neuro‐mesenchymal* miRNA subclasses contained a mix of samples from all four mRNA subclasses. Among the clinical features investigated by the authors, it was interesting to notice that when analysing the clinical response to radiation and temozolomide combination treatment, it appeared that tumours from the *astrocytic subclass* have a significant survival benefit, even though the most significant association between *MGMT* promoter methylation and longer survival was observed in the *neuro‐mesenchymal subclass*.

Subclassification of glioblastoma based on the miR21‐*SOX2* regulatory axis, which is not captured by gene expression‐based classification, has been described as phenotypically, molecularly, and prognostically significant [106]. Using the median cutoff method, about 50% of 279 TCGA glioblastoma patients as well as 69 in‐house patients (M.D. Anderson Cancer Center, Houston, TX, USA) have been classified into two extreme subtypes: *Class A* and *Class B*. The remaining 50% of cases fall in between these two classes and have been defined as *Class C*. *Class A* is a progenitor‐like subclass, characterised by the signature miR21^High^/*SOX2* ^Low^, whilst the signature of *Class B* is miR21^Low^/*SOX2* ^High^ and it defines a stem‐like differentiation. *Class C* includes patients with miR21^Low^/*SOX2* ^Low^ and miR21^High^/*SOX2* ^High^ signatures. Patients in *Class B* showed significantly longer survival when compared to *Class A*. It is worth mentioning, however, that these findings are certainly confounded by the inclusion in *Class B* of astrocytomas IDH‐mutant CNS WHO grade 4 samples, which are known to have longer survival than IDH‐wildtype glioblastomas. Enrichment for inflammatory and immune‐response pathways were found in *Class A* when performing GSEA analysis, whilst *Class B* was enriched for CNS development pathways, a finding which could also be influenced by the inclusion of IDH‐mutant tumours. Importantly, though, neither *Class A* or *Class B* showed enrichment for a specific mRNA subtype [105].

Despite the lack of consistency between mRNA and miRNA‐based classifications, miRNAs were identified that discern mRNA glioblastoma subtypes. For example, miR23a, miR27a, and miR9‐3p are capable of distinguishing between *GSf‐like* and *GSr‐like‐proneural‐like* and *mesenchymal‐like,* respectively‐GICs as well as proneural and mesenchymal subtypes of glioblastomas in the TCGA data [107, 108]. MiR23a and miR27a were significantly upregulated in both *GSr‐* and mesenchymal patients compared to *GSf‐* and proneural tumours, while the opposite pattern was observed for miR9‐3p. Interestingly, when these three miRNAs were used as prognostic factors for all glioblastoma TCGA samples, irrespective of the mRNA‐based classification, they effectively classified patients into two distinct groups: *GSf‐like* and *GSr‐like,* with the latter having a significantly worse prognosis. The bidirectional transition between these two states appears to be also regulated by miRNAs, in particular by miR‐128, which acts through remodelling of the PRC‐complex [109].

Schneider *et al*. [110] exploited the amplification of the receptor tyrosine kinase *EGFR* to classify 80 glioblastoma patients into two subgroups: *EGFR‐amp* and *EGFR‐normal*. By analysing the landscape of miRNAs in this context, they found that miR‐182‐5p, miR‐96‐5p, and miR‐183‐5p were expressed at a significantly higher level in *EGFR‐amp* tumours. Interestingly, one of the main targets of this miRNAs cluster is *FOXO1*, a pro‐apoptotic gene, which appeared to be repressed in *EGFR‐amp* tumours.

MiR‐379/miR‐656 cluster (C14MC) is the second‐largest cluster of miRNAs and is located on human 14q32, which is a locus known to be unstable in gliomas [36]. It has been shown that this cluster is prognostically relevant in 350 glioblastomas from TCGA, given that it is expressed at a higher level in patients with better prognosis [111]. Additional miRNAs with prognostic value include miR‐181d, which represses the *MGMT* gene, hence functioning as a predictive biomarker of poor survival for glioblastoma patients upon TMZ treatment [112, 113] and miR‐182, which is associated with TMZ susceptibility and a more favourable prognosis [114]. Moreover, Yuan *et al*. [115] analysed 563 glioblastoma patients from TCGA and described a 3‐miRNAs (hsa‐miR‐222, hsa‐miR‐302d, and hsa‐miR‐646) signature with overexpression of hsa‐miR‐222, together with downregulation of both hsa‐miR‐302d and hsa‐miR‐646as as predictive of patients' prognosis. An additional signature comprising a combination of four miRNAs (hsa‐let‐7a‐5p, hsa‐let‐7b‐5p, hsa‐miR‐615‐5p, and hsa‐miR‐125a‐5p) and the methylation status of *MGMT* has been shown to effectively stratify 102 glioblastoma patients into two distinct risk groups: low‐risk (54.9% of patients) and high‐risk (45.1% of patients) [116]. In addition, a multivariate Cox analysis taking also patients age into account (≤ 60 or > 60 years) demonstrated that these three factors significantly predicted overall survival, with the inclusion of age/*MGMT* methylation enhancing the accuracy of the prediction. The authors further investigated the impact of the four miRNAs and DNA methylation on gene expression by integrating the datasets from 23 patients and reporting the following associations: for hsa‐miR‐125a‐5p genes *MDM4*, *PTEN*, *SND1*, *WT1*, *BRCA1*, *LMO2*, *ESR1*, *DNM2*, *MLLT10*; for hsalet‐7a‐5p genes *SF3B1* and *PTEN* and for hsa‐miR‐615‐5p the *CSF1R* gene. Finally, an additional three miRNAs (hsa‐miR‐20a, hsa‐miR‐21 and hsa‐miR‐10a) signature was published recently [117], whereby 37 patients were subclassified into two groups, high‐risk (58% of patients) and low‐risk (42% of patients) with the OS being lower and higher than 12 months, respectively. All three miRNAs have an oncogenic potential when overexpressed, with hsa‐miR‐20a promoting cell growth, invasion, and angiogenesis, hsa‐miR‐10a being associated with epithelial‐to‐mesenchymal‐transition (EMT), and hsa‐miR‐21 affecting radio/chemosensitivity and invasion potential.

### 2.4 lncRNA

Long noncoding RNAs belong to the wider group of noncoding RNAs and are characterised by a length of ≥200 bp. They contribute to the regulation of all major biological processes through regulation of gene expression at the epigenetic, transcriptional and post‐transcriptional level [118, 119, 120, 121, 122, 123, 124]. These molecules can perform their functions via several mechanisms of actions (Fig. 1C), including molecular signals [125], molecular decoys [126], molecular guides [127], scaffolds [128], competing endogenous RNA (ceRNA) [129], and enhancers [130]. LncRNAs have emerged as significant contributors to brain tumour pathogenesis, as they can have oncogenic or tumour suppressive potential, hence could represent actionable targets for precision diagnostic and personalised medicine [131].

**Fig. 4:**
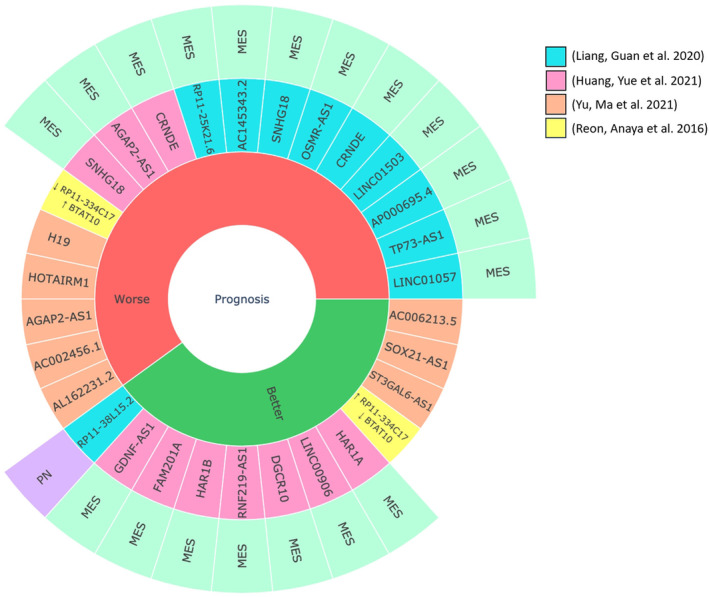
Sunburst plot of prognostic lncRNAs and their association with transcriptional subtypes. Long noncoding RNAs (lncRNAs) can be classified as associated with better or worse prognosis in glioblastoma patients. Each lncRNA is colour‐coded to represent the study where it was identified. The outer layer of the plot is depicting any association between each lncRNA and a transcriptional glioblastoma subtype.

In 2016, by comparing transcripts from 170 glioblastoma samples and 78 normal brain samples, 64, 211, 95 and 71 lncRNAs were identified that were specifically deregulated in neural, proneural, mesenchymal, and classical glioblastoma subtypes, respectively, demonstrating that expression profiles are highly distinct between subtypes [132]. To assess the prognostic value of these signatures, a multivariate Cox regression analysis was carried out which identified 584 and 282 lncRNAs associated with a poor or better prognosis, respectively. High expression of *RP11‐334C17* and low expression of *BTAT10* characterise patients with a median survival of 485 days [132], while low expression of *RP11‐334C17* and high expression of *BTAT10* is found in patients with poorer survival (380 days and 335 days, respectively) (Fig. 4). Interestingly, lncRNAs associated with a poor prognosis were found to be associated with cell cycle pathways, chromosome organisation, and immune response, whilst signalling pathways, signal transduction pathways, and phosphorylation pathways were found in patients with a better prognosis. To explore whether an association could be found between subtype‐specific lncRNAs and prognosis, a Cox regression was performed for each lncRNA in a given subtype, identifying 88, 385, 128, and 165 lncRNA molecules specifically in neural, proneural, mesenchymal, and classical glioblastoma, respectively. Within these subtype‐specific prognostic lncRNAs, 29, 87, 70, and 117 molecules were associated with poor prognosis. Very little overlap was found between subtypes, confirming that the lncRNA expression profile varies substantially between patients; a conclusion supported also by another study [133], where analysis of 500 lncRNAs with the least coefficient of variation between five glioblastoma tumours identified only 175 shared between all samples. In contrast, a recent study, which integrated the TCGA‐GBM expression profile and RNA interaction data to obtain lncRNA‐mediated ceRNA (LMce) networks in the different transcription‐based glioblastoma subtypes, found that the majority of the activated mRNAs and lncRNAs were the same. However, their regulation at the ceRNA level was substantially different between glioblastoma subtypes with 42.5%, 50.9%, 43.5%, and 65.5% of all lncRNA‐mRNA regulations being specific for classical, mesenchymal, proneural, and neural, respectively. Considering how lncRNAs and mRNA synergise with each other within each subtype, a final number of 61, 132, 24, and 16 synergetic lncRNA‐mRNA competitive modules was found for CL, MES, PN, and NE, respectively. Different GO terms identified taking into account synergies between lncRNAs and mRNA within each subtype identified modules that could be used to find novel prognostic biomarkers [134].

Interestingly, four glioblastoma subtypes (A, B, C, D) have been identified from 168 glioblastoma samples upon screening 17 immune‐related lncRNA (irlncRNA) by multivariate Cox regression analyses to build an lncRNA prognostic model. Subtype A comprises 23 cases and has the most favourable prognosis, perhaps not surprising, given that it includes four cases of IDH‐mutant grade 4 astrocytoma. These patients had low expression levels of high‐risk lncRNAs (*H19*, *HOTAIRM1*, *AGAP2‐AS1*, *AC002456.1*) and one high‐risk gene (*KRT8*). *H19*, *AL162231.2*, *AC002456.1,* as high‐risk factors, as well as *ST3GAL6‐AS1*, *SOX21‐AS1*, *AC006213.5* as protective factors were also identified in this study (Fig. 4). Taking advantage of a ceRNA network constructed to predict lncRNA targets, PLAU (Urokinase‐Type Plasminogen Activator) was identified as a prognostic biomarker overexpressed in cancer‐related pathways (in KEGG pathway analysis), which is the target of lncRNA *H19* via miR‐193‐3p [135]. This gene has indeed been described as upregulated in glioblastoma [136], where it plays a role in the promotion of cell invasion via PLAUR (PLAU Receptor) [137].

The mesenchymal subtype of glioblastoma is the most aggressive and it is the most represented subtype at recurrence [138]. It is the subtype more resistant to therapy [139] and it relies on both intrinsic and extrinsic TME‐related mechanisms to acquire and maintain the mesenchymal state [140]. LncRNAs influence this process and, for example, the binding of the MES‐related lncRNA *miR155HG* to miR‐185 promotes the epithelial‐mesenchymal transition and influences apoptosis, proliferation, and cell cycle progression [141]. Moreover, the MES‐related lncRNA *FAM181A‐AS1* enhances *ZRANB2* expression, and as such promotes both survival and growth of glioma cells [142]. MES‐related lncRNAs signatures have been used recently for prognosis assessment [143, 144] (Fig. 4), with the majority of the lncRNAs being a predictor of poor prognosis and the high‐risk group being predominantly composed of mesenchymal cases [144]. It is interesting to note that CRNDE and SNHG18 were identified as markers of poor prognosis by both the latter studies.

A recent classification of glioblastoma based on single‐cell lncRNA expression profiles and subsequent validation on TCGA data classified this cancer at the tissue level, based on the dominant number of cells belonging to a certain subtype despite the high intratumour heterogeneity [145]. Four new subtypes were identified, each characterised by a specific marker lncRNA: (I) *LINC00273*, (II) *LINC00461*, (III) *LINC00339*, (IV) *MEG3*. Integration with three prognostic groups identified from 152 TCGA cases, referred to as *population 1*, *2,* and *3*, and whose prognostic order from best to poor is *3*, *1*, *2*, showed that *population 3*, the one with the best prognosis, is characterised by a higher expression of type IV, but also types II and III. *Population 1* mainly expressed markers of type II and III and, finally, *population 2*, the one with the worst survival, was mainly expressing markers of type III. Thus, glioblastoma with a worse prognosis tend to exhibit greater cellular subtype purity.

## 3 Glioblastoma ontogeny and its contribution to intertumour heterogeneity

It is intriguing to speculate that ontogeny, in other words, the cell of origin, contributes to intertumour heterogeneity in glioblastoma. In fact, the many parallels between epigenetic diversity across cell types and developmental stages during brain development and glioblastoma subtypes support this interpretation.

Deep sequencing of somatic mutations in 97 tumour‐free samples from the subventricular zone (SVZ), one of the main areas where NSC resides in the adult brain, from 30 glioblastoma patients, revealed driver mutations in the NSC, suggesting that this population is the cell of origin of at least a proportion of these tumours [146]. In keeping with this conclusion, *PTEN*‐deficient human NSC undergo malignant transformation when injected into the brain of immunocompromised NOD/SCID mice, giving rise to tumours expressing *SOX2*, *GFAP*, *NESTIN*, *TUJ1*, *MAP2,* and *CD133*, similarly to human glioblastoma [147]. In the mouse, inactivation of *Pten* and *Trp53* in NSC using the Cre‐LOXP system led to their malignant transformation into high‐grade gliomas [148]. Importantly, other progenitor cells may also act as a glioblastoma cell of origin and, in fact, the gene expression signatures enriched for in the three transcriptional subtypes (CL, PN, and MES) recapitulated signatures of defined neural lineages, neural progenitor cells (NPC)/ astrocyte progenitor cells (APC), NPC/oligodendrocyte progenitor cells (OPC), and astroglial/immune, respectively [105]. *In vivo* experiments taking advantage of cell‐specific expression of the Cre recombinase, demonstrated that not only NSC [149] but also *Ascl1* ^+^ adult bipotential (neural and oligodendrocyte) progenitors and *Ng2* ^+^ adult OPC [150] led to glioma formation when the tumour suppressor *Nf1*, *Trp53,* or *Pten* were inactivated. Interestingly, two distinct types of tumours developed in the compound mutants: type 1 (infiltrative, *G*fap^high^) and type 2 (circumscribed, Gfap^low^), with bipotential progenitors giving rise to both types, whilst adult OPC gave rise only to type 2, indicating that different cells of origin give rise to different tumour subtypes. Notably, not all progenitors can be transformed and *Neurod1* ^+^ newly‐born neurons, *Dlx1* ^+^ late‐stage neuronal progenitors, and *Camk2a* + postmitotic differentiated neurons did not give rise to tumours [151].

Epigenetic deregulation certainly contributes to ontogeny, being a driver of diversity in glioblastoma. In human GIC, a hypomethylation bias was observed in a proportion of lines that affected binding motifs of TFs linked to astrocyte differentiation; concomitant deregulation of the expression of miRNAs involved in glial lineage specification suggested that these GICs may originate from APC [152]. Chromatin accessibility profiles of mouse GIC cultures derived from transgenic mice overexpressing the oncogene *PDGFB* under the control of promoters specific for NSC, OPC, or APC were different and depended on the cell of origin. Importantly, clusters that showed overlapping characteristics with each of the mouse groups were identified when 60 human GIC cultures were analysed [84], hence highlighting the translational value of the observation.

Models that faithfully recapitulate the epigenetic contribution to the intertumour heterogeneity in glioblastoma are now required to ensure it can be harnessed for precision medicine. Patient‐derived glioblastoma organoids (GBOs), GLICOs, and patient‐derived xenografts (PDXs) have been shown to recapitulate, to a reasonable extent, the histological architecture, genetics, and transcriptome of patients' tumours and are being extensively applied at the preclinical and translational level [153, 154, 155, 156]. Importantly, confirmation of their value also as models of patient‐specific epigenetic deregulation is emerging, as shown using the GLICO model. Here patient‐specific chromatin states were detected, and GIC resembling radial glial‐like cells and early stem‐like states sharing similar trajectories with the original tumour were identified in all patients examined [96]. PDXs derived from 96 glioblastoma patients recapitulated phenotypic and molecular characteristics of the original tumour, not only at the genetic and transcriptional levels, but also at epigenetic levels, as assessed by genome‐wide DNA methylation profiling [156].

To leverage any patient‐specific epigenetic change for precision medicine in glioblastoma, tools that allow drug matching are required, predicting the effectiveness against the tumour cells, particularly GIC, which are at the apex of the tumour hierarchy. The challenge, though, is that cells of origin are not readily available for comparison with GIC. NSC populations from foetal brain [157] have been used, although syngeneic ones would be a more powerful comparator from a precision‐medicine perspective. Harnessing state‐of‐the‐art cell reprogramming technology, expanded potential stem cells (EPSC)‐induced NSC and GIC pairs (SYNGN) were derived for 10 patients and were shown to be a suitable platform to identify druggable target genes at the preclinical level [158].

## 4 Outlook

It is striking that despite the considerable advances in our understanding of the genetic basis of glioblastoma onset and recurrence [6], no effective therapies have been developed in the past two decades and survival has not improved. Heterogeneity is certainly a major challenge in glioblastoma, where it significantly contributes to tumour diversity between patients and to plasticity and adaptive evolution in individual patients. As described in this review, epigenetic deregulation significantly contributes to the prominent cellular and molecular heterogeneity of these tumours and it will be essential to take it into account in the design of future clinical trials. To date, clinical trials integrating personalised therapeutic strategies alongside standard‐of‐care treatments in glioblastoma have focussed on targeting genetic events or, more recently, on leveraging autologous dendritic cell vaccines [2], but none are targeting epigenetic mediators. This is in stark contrast to paediatric high‐grade brain tumours, such as medulloblastoma (MB) and diffuse intrinsic pontine gliomas (DIPG) [36, 159, 160]. In fact, elucidation of deregulated epigenetic mechanisms led to a profound impact on subgroup classification, prognosis, and response to therapy in the former tumour and clinical trials, for the latter neoplasms are currently exploiting epigenetic drugs, mainly targeting aberrant DNA methylation and histone modifications [159].

It is also evident, however, that aberrant epigenetic mechanisms are complex in glioblastoma and impacting multiple regulatory layers; hence, integration of profiles will be key for a better understanding of the global molecular landscape of the tumour. Although integration of omics data remains challenging, artificial intelligence methodologies are currently being explored to enable this integrative analysis, which may refine subgroup classification, as well as enable the identification of druggable targets in each subgroup or at the patient‐specific level [161]. For instance, neural networks have been used to create an *in‐silico* patient‐specific drug repurposing tool, which is based on genetic and transcriptomic profiles [162]. Similarly, the Drug–Gene Interaction Database (DGIdb) can be used to identify compounds known to interact with predicted targets for drug repurposing [163]. It will also be important to harness novel technologies enabling the characterisation of epigenetic changes at single‐cell resolution to tackle intratumour heterogeneity, thus ensuring the cellular hierarchy of glioblastoma is taken into account and specific vulnerabilities of cancer stem cells are identified and targeted in future trials. Importantly, tools for targeting traditionally challenging targets for protein‐based therapies, including epigenetic regulators, are becoming available and, for example, RNA‐based treatment methods can be envisaged as an attractive option. In addition, advanced nanotheranostic approaches, which utilise biocompatible nanoparticles for targeted delivery, could be leveraged to enable cell‐specific delivery of novel agents.

## 5 Conclusion

In conclusion, epigenetic dysregulation significantly contributes to interpatient heterogeneity in glioblastoma, and it will be important to take this into account when defining the molecular landscape of the disease to achieve a more accurate subclassification and to develop matched precision therapy strategies.

## Conflict of interest

The authors declare no conflicts of interest.

## Author contributions

SL and SM conceptualised, wrote, and revised the article with contributions from MC for the DNA methylation and ontogeny chapters. SL and MC created the figures.
